# Mutual Inhibition between Kaposi's Sarcoma-Associated Herpesvirus and Epstein-Barr Virus Lytic Replication Initiators in Dually-Infected Primary Effusion Lymphoma

**DOI:** 10.1371/journal.pone.0001569

**Published:** 2008-02-06

**Authors:** Yanjun Jiang, Dongsheng Xu, Yong Zhao, Luwen Zhang

**Affiliations:** 1 Nebraska Center for Virology, University of Nebraska, Lincoln, Nebraska, United States of America; 2 School of Biological Sciences, University of Nebraska, Lincoln, Nebraska, United States of America; University of Hong Kong, China

## Abstract

**Background:**

Both Kaposi's sarcoma-associated herpesvirus (KSHV) and Epstein-Barr virus (EBV) are members of the human gamma herpesvirus family: each is associated with various human cancers. The majority of AIDS-associated primary effusion lymphoma (PEL) are co-infected with both KSHV and EBV. Dually-infected PELs selectively switch from latency to lytic replication of either KSHV or EBV in response to chemical stimuli. KSHV replication and transcription activator (K-RTA) is necessary and sufficient for the switch from KSHV latency to lytic replication, while EBV BZLF1 gene product (EBV-Z) is a critical initiator for induction of EBV lytic replication.

**Methodology/Principal Findings:**

We show K-RTA and EBV-Z are co-localized and physically interact with each other in dually-infected PELs. K-RTA inhibits the EBV lytic replication by nullifying EBV-Z-mediated EBV lytic gene activation. EBV-Z inhibits KSHV lytic gene expression by blocking K-RTA-mediated transactivations. The physical interaction between K-RTA and EBV-Z are required for the mutual inhibition of the two molecules. The leucine heptapeptide repeat (LR) region in K-RTA and leucine zipper region in EBV-Z are involved in the physical interactions of the two molecules. Finally, initiation of KSHV lytic gene expression is correlated with the reduction of EBV lytic gene expression in the same PEL cells.

**Conclusions/Significance:**

In this report, how the two viruses interact with each other in dually infected PELs is addressed. Our data may provide a possible mechanism for maintaining viral latency and for selective lytic replication in dually infected PELs, *i.e*., through mutual inhibition of two critical lytic replication initiators. Our data about putative interactions between EBV and KSHV would be applicable to the majority of AIDS-associated PELs and may be relevant to the pathogenesis of PELs.

## Introduction

Epstein-Barr virus (EBV) and Kaposi sarcoma (KS)-associated herpesvirus (KSHV), also called human herpesvirus 8 (HHV-8), are the two gamma herpesviruses currently identified in humans. EBV infection has been associated with the development of Buikitts' lymphoma (BL), Hodgkin's disease, nasopharyngeal carcinoma and others [1]–[5]. KSHV is believed to be the etiological agent of KS [6]–[8], and is implicated in the pathogenesis of AIDS-associated primary effusion lymphoma (PEL), also called body cavity-based lymphoma (BCBL), and multicentric Castleman's disease [6], [9], [10].

Like other herpesviruses, both EBV and KSHV have latency and lytic replication in their life cycles. The switch from latency to lytic gene expression in KSHV requires the expression of KSHV replication and transcription activator (K-RTA, also called RTA or ORF50). K-RTA is apparently necessary and sufficient for the switch from KSHV latency to lytic replication [9], [10]. K-RTA is a sequence-specific DNA-binding protein that regulates gene expression through K-RTA-responsive elements in the transcriptional regulatory regions of different subsequently expressed viral genes [11]–[18]. K-RTA also interacts with other factors to modulate its transcription potential, and some interactions are ctritical for K-RTA-mediated switch from latency to lytic replication [19]–[24].

Beyond functioning in initiating viral lytic replication, K-RTA is involved in the induction of cellular IL-6 [25]. K-RTA also blocks p53-mediated apoptosis by competing for binding to CBP [26]. K-RTA might play a role in latency establishment [27], [28]. K-RTA has been shown to enhance CD21 expression, and facilitate EBV infection because CD21 is EBV receptor in B cells [29].

EBV lytic replication can be initiated by expression of the EBV BZLF1 gene product (EBV-Z; also referred as BZLF1, Zta, Z, ZEBRA, and EB1) [30], [31]. EBV-Z is a member of the basic leucine zipper (bZIP) family of DNA binding proteins and has a sequence similar to C/EBP, c-Jun, and c-Fos [32]. EBV-Z binds specifically to DNA with multiple specific recognition sequences and activates transcription of both viral and cellular genes [33]–[36]. One important viral gene target of EBV-Z is EBV BRLF1 gene product, E-RTA (also called BRLF1, RTA, R). K-RTA and E-RTA are homologues genes and this family of genes is highly conserved among gamma herpesviruses. E-RTA is coordinately expressed as a bicistronic RNA transcript with EBV-Z [37]. E-RTA and EBV-Z function synergistically at some promoters and are required for the completion of the EBV lytic replication cycle [38].

EBV-Z has additional activities other than initiation of EBV lytic replication. These include the ability to block cell cycle progression [39], [40] and the disruption of the PML-associated nuclear domain 10 (ND10/PODs) [41], [42]. EBV-Z can also upregulate its own expression, a property called autoregulation [43], [44]. Of note K-RTA also auto-regulates its own expression [14], [45].

PELs are B-cell non-Hodgkin's lymphomas and most frequently occur in HIV-positive individuals as lymphomatous effusions in the serous cavities without a detectable solid tumor mass. In the setting of AIDS, the clinical course for most of these lymphomas is extremely aggressive, with a mean survival from diagnosis of 5–7 months [46]. While PELs are almost universally KSHV-positive, the majority of PELs have concomitant EBV infection [6], [10], [47].

In order to understand viral contributions to the pathogenesis of PEL, it is important to address how EBV and KSHV interact with each other and affect biological properties of the cell and the viruses. There are apparent interactions between the two viruses in PELs. EBV enhances the tumorigenecity of the dually-infected PELs in SCID mice [48]. Unique sets of cellular genes are expressed in dually-infected, but not single KSHV-infected PELs [49]. KSHV LANA potentially activates EBV latent membrane protein 1 (LMP-1) [50] , but reduces the expression of EBV EBNA-1 and represses EBV EBNA-2 activation [51]. K-RTA may potentiate EBV latency via induction of EBV LMP-1 and uses LMP-1 to curb KSHV lytic replication [52].

Of particular interest in dually infected PELs is the selective induction of KSHV or EBV lytic replication [12], [53]. BC-1 is a dually infected PEL line. EBV lytic gene expression is activated by 12-O-tetradecanoylphorbol-13-acetate (TPA or phorbol ester). KSHV lytic gene expression is induced by sodium butyrate (butyrate or n-butyrate). However, when both TPA and butyrate are added, KSHV, rather than EBV, is induced into lytic replication [12], [53]. The selective induction of lytic replications of the two herpesviruses points to the importance of viral factors in the decision-making processes of PELs.

In this report, we have examined the potential molecular mechanism behind this selective lytic replication process. We have found that there is a mutual inhibition between EBV-Z and K-RTA. Because of their critical roles of the two molecules in promoting their respective viral lytic replications, our data offer a possible mechanism for dually-infected cells to keep their respective viral latencies and for selective switch from latency to lytic replications.

## Results

### K-RTA negatively regulates the lytic gene expression of EBV

**Figure 1:**
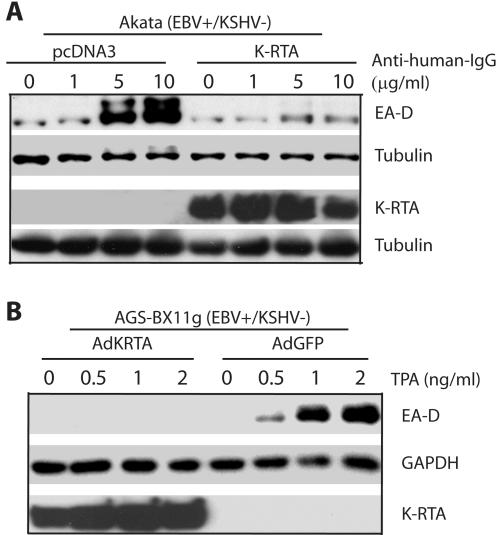
K-RTA inhibits EBV lytic gene expression. A. K-RTA inhibits the EBV lytic gene expression in Akata cells. Akata (EBV+/KSHV-) cells were transfected with K-RTA or vector plasmid (5 µg DNA). EBV lytic replication was induced by anti-human-IgGs a day later (0, 1, 5, 10 µg/ml). Cell lysates were separated on SDS-PAGE, transferred, and used for western blot analysis. The identity of proteins is as shown. B. K-RTA inhibits the EBV lytic gene expression in AGS-BX11g cells. AGS-BX11g (EBV+/KSHV−) cells were infected with recombinant adenovirus expressing K-RTA or GFP (10 pfu/cell). EBV lytic replication was induced by TPA a day later. Lysates were used for western blot analysis. The same membrane was stripped and reprobed with other antibodies. One representative from three experiments is shown. The identity of proteins is as shown.

Because of the co-presence KSHV and EBV in a same PEL cell and the selective induction of lytic replication, we suspect that one or more proteins in KSHV might regulate EBV lytic replication process. K-RTA is a good candidate because it is immediate early protein and able to activate an EBV gene [52]. We thus examined if K-RTA affect the induction of lytic replication of EBV. K-RTA was transfected into Akata cells, an EBV+/KSHV- BL line, and the lytic replication of EBV was induced after the treatment with anti-human-IgG (See Materials and Methods for details). EBV BMRF1 is a lytic gene and its product is often called the diffuse component of the EBV-early antigen (EA-D). The essential function of EA-D in EBV lytic replication has been well established and using it as indicator of lytic replication has been widely accepted and appreciated in the field [54]–[60]. Thus, the expression of EA-D protein was determined and used as indicator of EBV lytic replication. As shown in the Fig. 1A, the expression of EA-D was inhibited upon the expression of K-RTA. Of note multiple bands of EA-D are often observed during EBV lytic replication.

In addition, we tested if the same phenomenon can be observed in another cell line, AGS-Bx1g (EBV+, KSHV−). These cells were infected by either recombinant adenovirus expressing K-RTA (AdKRTA) or green fluorescent protein (AdGFP). One day after the infection, the lytic replication of EBV was examined by the treatment with TPA. As shown in Fig. 1B, induction of EA-D protein expression and furthermore the EBV lytic replication was inhibited by the expression of K-RTA. These data suggested that K-RTA was a negative regulator of EBV lytic gene expression.

### K-RTA negatively regulates EBV-Z initiated lytic gene expression of EBV

It is well established that EBV-Z initiates the lytic replication of EBV. We further examined that if K-RTA is able to inhibit the EBV-Z-mediated induction of EBV lytic gene expression.

**Figure 2:**
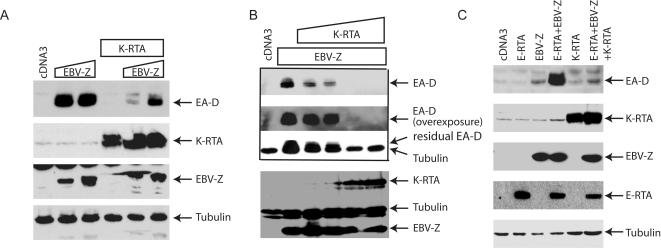
K-RTA inhibits EBV-Z-mediated EBV lytic gene expression. A. K-RTA inhibits EBV-Z-mediated EBV lytic gene expression. EBV-Z expression plasmid (0, 0.1, and 0.2 µg) plus K-RTA (0.2 µg) were transfected into BRLF1KO (EBV+/KSHV−) cells in 6-well plate as shown on the top. Lysates were used for western blot analysis 24 hours later. The same membrane was stripped and reprobed with other antibodies. The identity of proteins is as shown. B. Dose-dependent inhibition of EBV-Z-mediated lytic gene expression by K-RTA. Fix amount of EBV-Z expression plasmid (0.1 µg) plus various amounts of K-RTA (0, 0.05, 0.1, 0.2, 0.4 µg) were transfected into BRLF1-KO (EBV+/KSHV−) cells in 6-well plate as shown on the top. Lysates were used for western blot analysis. Same cell lysates were used. C. K-RTA inhibits the synergistic activation of EA-D. EBV-Z expression plasmid (0.025 µg), E-RTA (0.1 µg), and K-RTA (0.2 µg) were transfected with different combinations into BRLF1-KO cells as shown on the top. The same cell lysates were used for western blot analysis. The identity of proteins is as shown.

A serial of cell lines, 293-EBV, BRLF1-KO, and BZLF1-KO, was used for the experiments. 293-EBV harbors wild type EBV genome. BRLF1-KO and BZLF1-KO contain the EBV genome missing BRLF1 (E-RTA) or BZLF1 (EBV-Z) gene respectively [38]. BRLF1-KO was used primarily because expression of EA-D was not very sensitive to EBV-Z expression (data not shown). As shown in the Fig. 2, EBV-Z is able to induce the lytic replication of EBV as indicated by the induction of EA-D protein. However in the presence of K-RTA, the expression of EA-D is inhibited (Fig. 2A). The inhibition is dose-dependent phenomena (Fig. 2B). Interestingly, EBV-Z and E-RTA can synergistically induce EBV lytic replication [61]–[64]. By reducing the expression of EBV-Z with less plasmid in transfection, we could observe the reported synergy and K-RTA was a potent inhibitor of the synergistic activation (Fig. 2C). Essentially the same results can be obtained from 293-EBV and BZLF1-KO cell lines (data not shown). The use of more than one cell lines is to ensure the results are not cell-line dependent. These data suggested that K-RTA inhibited EBV-Z-mediated lytic gene expression.

### K-RTA and EBV-Z physically interact in dually-infected PEL

Next we examined the potential mechanism for K-RTA-mediated inhibition of EBV lytic replication. Because: 1) EBV-Z and E-RTA interact functionally and physically [61]–[64]; 2) K-RTA and KSHV K8, an EBV-Z homologue, also interact functionally and physically [22], [65], [66]; and 3) EBV-Z and KSHV K8 interact with same cellular genes, such as p53, CBP and C/EBPα [67]–[74], we hypothesize EBV-Z and K-RTA interact with each other physically.

**Figure 3:**
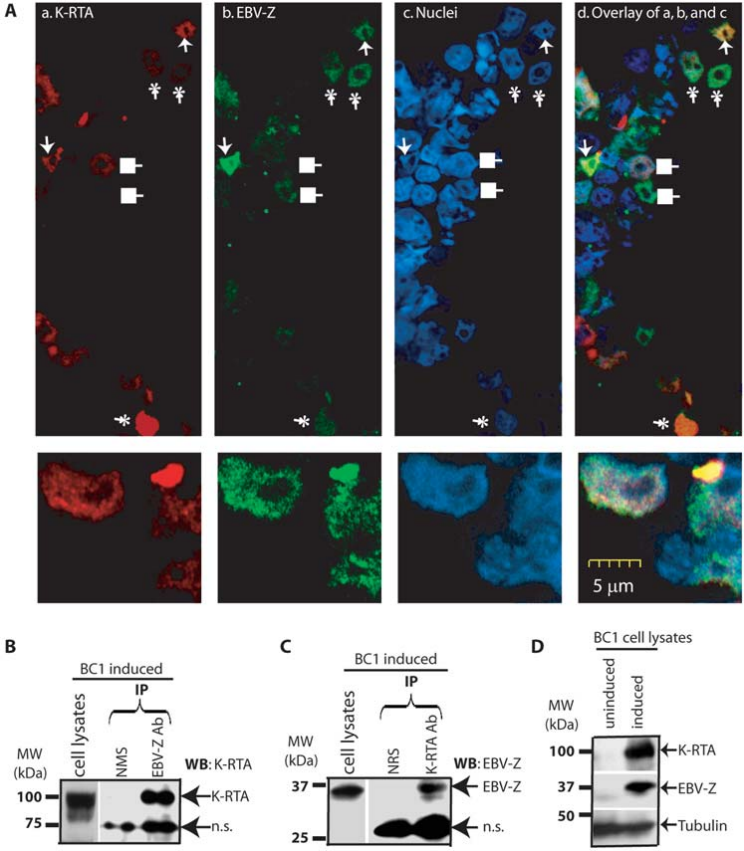
K-RTA and EBV-Z interact with each other in dually infected cells. A. Co-localization of K-RTA and EBV-Z in dually infected cells. BC1 (KSHV+/EBV+) cells were treated with TPA (10 ng/ml) first for one day and then butyrate (0.5 mM) for another day. Cells were fixed and stained with K-RTA (rabbit) and EBV-Z (mouse) antibodies. Cy5- and Cy2-labeled secondary antibodies were used to distinguish the signals from K-RTA and EBV-Z, respectively. DAPI was used to stain the nuclei. The colors were artificially mounted to facilitate viewing. Red, K-RTA; green, EBV-Z; blue, nuclei; (a)K-RTA signal only; (b) EBV-Z signal only; (c) nuclei only; (d) K-RTA, EBV-Z, and nucleus signals are mixed. The pictures of higher power are shown on the bottom. In Panels B and C, cell extracts from treated BC1 cells were immunoprecipitated (IP) with either anti-EBV-Z or normal mouse serum (Panel B). Cell lysates were also immunoprecipitated with anti-K-RTA or normal rabbit serum (Panel C). The immunoprecipitates were analyzed by Western blot using the indicated antibodies. The whole cell lysates of induced BC1 cells were used as positive controls in Panels B and C. In Panel D, whole cell lysates was used for western blot analyses. The identity of the respective proteins is denoted. n.s., non-specific. Molecular weight (MW) makers are shown on the left in kilo-Dalton (kDa).

BC1 (EBV+, KSHV+) cells were treated with TPA first to initiate EBV lytic replication and then treated with butyrate for KSHV lytic replication. The cells were fixed and stained with both K-RTA and EBV-Z antibodies. The localization of K-RTA and EBV-Z was examined under confocal microscope. As shown in the Fig. 3A, many cells contain both K-RTA and EBV-Z in the same nuclei (arrows and asters). Some K-RTA and EBV-Z may be co-localized in the same nuclei (see arrows; yellow color in the Panel d); suggesting they might be interacting with each other physically. The high powered versions of the cells are also present at the bottom. However in some of the cells, both EBV-Z and K-RTA are expressed in the same cells but the co-localization is not apparent, possibly due to the fact that one protein is expressed at much higher levels than the other (see asters). Some cells express either K-RTA or EBV-Z (see solid squares). Thus EBV-Z and K-RTA can be co-expressed and co-localized within dually infected cells.

Next, the co-immunoprecipitation assays were used for detection of potential physical interactions between K-RTA and EBV-Z in the induced BC-1 cells. Cell lysates were used for immunoprecipitation with either K-RTA or EBV-Z antibody. The immunoprecipitates were then used for western blot analysis with other specific antibodies. As shown in Fig. 3B, EBV-Z antibody could bring down K-RTA protein. In addition, the K-RTA antibody could bring down EBV-Z protein (Fig. 3C). However, neither normal rabbit serum (NRS) nor normal mouse serum (NMS) could precipitate EBV-Z or K-RTA protein (Fig. 3B, 3C). The induced cells express both K-RTA and EBV-Z proteins (Fig. 3D). These data suggest that EBV-Z and K-RTA interact with each other in induced BC1 cells in vivo.

### Interaction with EBV-Z is required for K-RTA-mediated inhibition of EBV lytic replication

**Figure 4:**
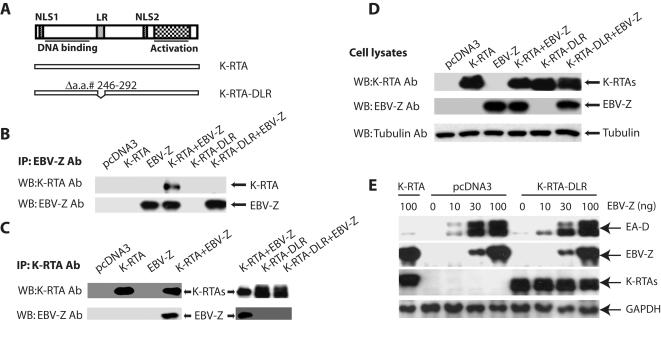
Interaction between K-RTA and EBV-Z is required for K-RTA-mediated inhibition. A. Schematic diagram of K-RTA domains and mutants. The DNA binding domain, leucine heptapeptide repeat region (LR), activation domain, and nuclear localization signal (NLS) are shown. The drawing is not on scale. In Panels B, C, and D, 293T cells were transfected with of the designated expression plasmids as shown on the top of the Figure. Cell extracts from these transfected cells were immunoprecipitated with either anti-EBV-Z (Panel B) or anti-K-RTA (Panel C). The immunoprecipitates were analyzed by Western blot (WB) using the indicated antibodies. In Panel D, whole cell lysate was used for western blot analyses. The identity of the respective proteins is denoted. E. Interaction between K-RTA and EBV-Z is required for K-RTA-mediated inhibition. 80 ng of K-RTA or K-RTA-DLR expression plasmids were transfected with various amounts of EBV-Z expression plasmid into BZLF1-KO (EBV+/KSHV−) cells as shown on the top. Lysates were used for western blot analysis 24 hours later. The same membrane was stripped and reprobed with other antibodies. The identity of proteins is as shown.

We suspect that K-RTA may physically interact with EBV-Z through its leucine heptapeptide repeat region (LR) of K-RTA [75]. This region is included within the domain of K-RTA required for interaction with the several cellular proteins such as K-RBP, RBP-Jκ, and C/EBPα [19], [23], [76], [77]. A mutant with the deletion of the region, K-RTA-DLR, was generated (Fig. 4A). The mutant protein is localized predominantly in the nucleus as wild type K-RTA (data not shown). The plasmids expressing EBV-Z, K-RTA and its mutant were transfected into 293T cells, and the interaction between EBV-Z and the mutant K-RTA was examined. While wt K-RTA interacted with EBV-Z properly, the K-RTA-DLR failed to interact with EBV-Z (Fig. 4B, and 4C). The expression of these proteins in 293T cells were similar (Fig. 4D). Thus, the LR region of K-RTA was involved in the physical interaction with EBV-Z.

Whether the physical interaction is involved in the repression of EBV-Z-mediated EBV lytic gene expression was examined in BZLF1-KO (EBV+, KSHV−) cell line. While wt K-RTA was able to repress EBV lytic replication as expected, the K-RTA-DLR mutant failed to inhibit the expression of EBV-Z-mediated EA-D expression (Fig. 4E). Same results can also obtained from 293-EBV (EBV+, KSHV−) cell line (data not shown). Thus, the interaction between K-RTA and EBV-Z was required for K-RTA-mediated inhibition of EBV lytic gene expression.

### EBV-Z inhibits the lytic gene expression of KSHV

**Figure 5:**
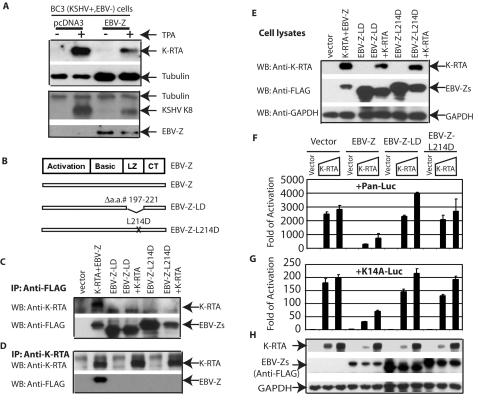
EBV-Z inhibits KSHV lytic gene expression. A. EBV-Z inhibits KSHV lytic gene expression. BC3 (KSHV+/EBV−) cells were transfected with CD4 expressing plasmid along with EBV-Z or vector plasmids. The transfected cells were isolated and equally split into two wells: one well of the cells was treated with TPA for 24 hours. Cell lysates were used for western blot analysis. B. Schematic of EBV-Z functional domains and mutants. The activation domain, basic region (DNA binding domain), leucine zipper region (LZ), and a region of unknown structure at the C terminus (CT) are shown. The drawing is not on scale. In Panels C, D, and E, 293T (EBV−/KSHV−) cells were transfected with various expression plasmids as shown on the top. FLAG-EBV-Z, and its mutants were used. Cell extracts from these transfected cells were immunoprecipitated with either anti-FLAG (for EBV-Z) (Panel C) or anti-K-RTA (Panel D). The immunoprecipitates were analyzed by Western blot using the indicated antibodies. In Panel E, whole cell lysate was used for western blot analyses. The identity of the respective proteins is denoted. In Panels F, G, and H, 293T (EBV−/KSHV−) cells were used. Panel F, KSHV Pan-promoter reporter construct (Pan-luc) and CMV-β-gal expression plasmid were cotransfected with 400 ng of EBV-Z or its mutant expression plasmids, together with 0, 20, 50 ng of K-RTA expression plasmids respectively as shown on the top. In Panel G, KSHV K14-promoter reporter construct (K14A-luc) and CMV-β-gal expression plasmid were cotransfected with 100 ng of EBV-Z or its mutant expression plasmids, together with 0, 10, 20 ng of K-RTA expression plasmids respectively as shown on the top. Luciferase activity was normalized by β -galactosidase activity. The relative folds of activation of promoter constructs are shown with standard deviations. One representative of three independent experiments is shown. Panel H, cell lysates from Panel F were used for western blot analysis. The same membrane was stripped and reprobed with other antibodies. The identity of proteins is as shown.

Next, we examined if EBV-Z affected the induction of lytic gene expression of KSHV. EBV-Z was transfected into BC3 cells, a KSHV+/EBV− PEL line, and the lytic gene expression of KSHV was examined. Tranfected cells were enriched and split into two wells: one of which was treated by TPA (see Materials and Methods for detail). As shown in the Fig. 5A, the expression of K-RTA and KSHV K8, an early KSHV lytic gene, were inhibited upon the expression of EBV-Z. Due to well-established functions of K-RTA and K8, the results also suggested that EBV-Z inhibited lytic replication of KSHV.

### Interaction is required for EBV-Z-mediated inhibition of K-RTA transactivation

We suspect that EBV-Z uses its leucine zipper domain for the interaction with K-RTA (Fig. 5B). The deletion mutant of leucine zipper (LZ) domain was made (EBV-Z-LD). In addition, another point mutation within the domain (EBV-Z-L214D) was also made because the specific mutation was known to block the dimerization and functions of EBV-Z [78]. Both mutants are localized in the nucleus as wild type EBV-Z (data not shown).

The plasmids expressing EBV-Z or its mutants along with K-RTA were transfected into 293T cells. The interaction between EBV mutants and K-RTA was examined. Both mutants, EBV-Z-LD and EBV-Z-L214D failed to interact with K-RTA as shown in Fig 5C, 5D. The expression of these proteins in 293T cells was proper (Fig. 5E). Thus, the leucine zipper domain of EBV-Z, and Leucine 214 in particular, was involved in the interaction with K-RTA.

Next, we examined if these EBV-Z mutants could affect the functions of K-RTA. KSHV Pan and K14 promoter reporter constructs are potently regulated by K-RTA, and the transactivation was inhibited by the co-expression of EBV-Z. However, the expression of EBV-Z mutants failed to repress the transactivation functions of K-RTA (Figs. 5F, 5G). The expression of K-RTA, EBV-Z, and EBV-Z mutants was confirmed (Fig. 5H). These data suggested that the interaction between the EBV-Z and K-RTA was required for the inhibition of K-RTA mediated transactivation.

### Initiation of KSHV lytic gene expression correlated with the reduction of EBV lytic gene expression

BC-1 (EBV+, KSHV+) is known to induce EBV lytic replication upon the treatment of TPA. We have tested if we could change the outcomes of the lytic gene expression by alternation of TPA dosages.

**Figure 6:**
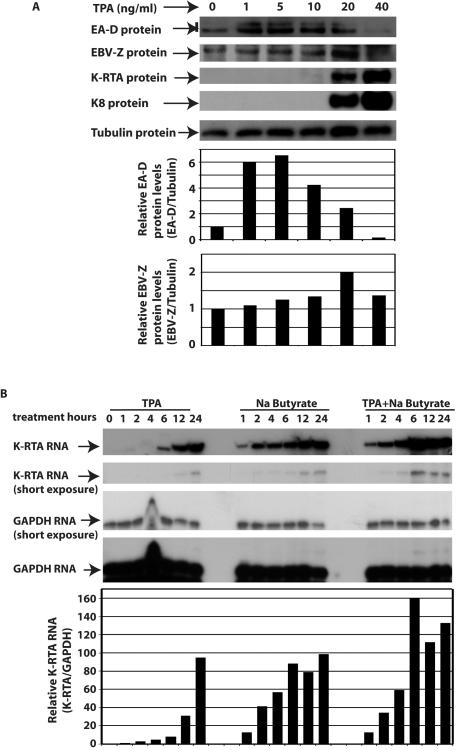
Initiation of KSHV lytic gene expression correlated with the reduction of EBV lytic gene expression. A. TPA induces either EBV or KSHV lytic replication. BC1 (EBV+/KSHV+) cells were treated with TPA at indicated dosages shown on the top. Cell lysates were used for western blot analyses a day later. The same membrane was stripped and reprobed with other antibodies. The identity of proteins is as shown. The relative levels of EA-D expression (EA-D/Tubulin) and EBV-Z (EBV-Z/Tubulin) were obtained by measuring intensity of EA-D, EBV-Z, and Tubulin using ImageJ 1.37v software (NIH), and are shown on the bottom panels. One representative from three independent experiments is shown. B. Kinetics of K-RTA expression in BC1 cells. BC1 cells were treated with TPA (20 ng/ml), or butyrate (3 mM), or both. Total RNA were isolated at indicated time post treatment. The expression of K-RTA and GAPDH RNA was monitored by RPA with K-RTA and GADPH probes simultaneously. Specific protections of K-RTA and GAPDH RNAs are indicated. The relative levels of K-RTA RNA expression (K-RTA/GAPDH) were obtained by measuring intensity of K-RTA and GAPDH using ImageJ 1.37v software (NIH), and are shown on the bottom. One representative from three independent experiments is shown.

BC1 cells were treated with different concentration of TPA, and the lytic gene expression of both EBV and KSHV were analyzed simultaneously. At low level of TPA treatments, EBV lytic gene expression was induced as indicated by the expression of EA-D as well as EBV-Z. However, at higher levels of TPA treatment, EBV lytic gene expression was turned down. Coincidently, the KSHV lytic gene expression was initiated as indicated by the expression of K-RTA and K-8. Thus, TPA could induce either EBV or KSHV lytic gene expression in BC cells depending on the dosages. More importantly, the KSHV lytic gene expression apparently correlated with dampened EBV lytic gene expression. EBV-Z was induced in a dose dependent manner. At TPA 20 ng/ml, EBV-Z was expressed at the highest levels, but the EA-D expression was dropped (Fig 6A; bottom panel). Based on the data in Figures 1 and 2, the expression of K-RTA might block the function of EBV-Z, which reduced the EA-D levels. Furthermore, the excess K-RTA might activate KSHV lytic replication as determined by KSHV K8 expression. It is of note that although different batches of TPA had different dose response curves, the general trend was the same even with TPAs from different companies (data not shown).

Finally, we examined kinetics of the K-RTA RNA expression under different induction conditions. BC1 cells were treated with different kinds of chemical inducers. As shown in Fig. 6B, butyrate or butyrate plus TPA could induce significant K-RTA RNA after 1-2 hours of treatments; however, TPA was able to induce the similar levels of expression only after 6 hours of the treatment. Thus comparing to butyrate or TPA plus butyrate, treatment with TPA induces delayed expression of K-RTA RNA in BC1 cells.

## Discussion

The mechanisms of latency and lytic replications of gamma herpesviruses are extensively studied. The induction processes of lytic replications are apparently heterogeneous and depend on the cell, virus, and inducing agent. The co-existance of both EBV and KSHV in majority of PELs is providing a unique opportunity to study the interaction between the two viruses and the potential role in the pathogenesis. The two viruses are apparently able to interact with each other as well as the host [49]–[52], and the presence of both viruses is more potent to promote tumor formation in rodent model [48].

In this report, we have provided evidence that the critical lytic replication initiators of KSHV and EBV interact at molecular levels. First, K-RTA and EBV-Z are co-localized, and the two proteins physically interact with each other in the same PEL cells in vivo (Fig. 3). Second, K-RTA inhibits the chemically-induced EBV lytic gene expression (Fig. 1), and the inhibition may be related to the fact that K-RTA inhibits EBV-Z mediated lytic replication process (Fig. 2). EBV-Z and R-RTA physically interact and synergistically activate EBV lytic gene expression. This synergistic activation of EBV lytic gene expression is also inhibited by K-RTA (Fig. 2C), which clear shows the functional difference between K-RTA and E-RTA. Third, EBV-Z has the similar effect on KSHV: EBV-Z inhibits the chemically-induced KSHV lytic gene expression processes (Fig. 5A), and the inhibition is likely due to that EBV-Z inhibits K-RTA-mediated transactivation (Fig. 5B–5H). Fourth, the physical interaction between K-RTA and EBV-Z is apparently required for the mutual inhibition of the two molecules (Figs. 4 and 5). The leucine heptapeptide repeat region in K-RTA and leucine zipper region in EBV-Z molecules are involved in the physical interactions between the two molecules (Figs. 4 and 5). Fifth, we have shown that initiation of KSHV lytic gene expression correlated with the reduction of EBV lytic gene expression (Fig. 6). All these data suggest that K-RTA and EBV-Z are physically interact and mutually inhibits each others' functions. However, we cannot exclude the possibility that K-RTA and EBV-Z proteins are interacting indirectly through other proteins, and we are investigating the subject currently. Finally, dually infected cells can express both K-RTA and EBV-Z in the same cells under the special induction conditions (Fig. 3A). Of note under routine induction conditions such as shown in Figure 6, immunostaining of EBV-Z and K-RTA showed that the majority of cells were either K-RTA or EBV-Z positive, and only a small fraction were positive for both (data not shown). All these data collectively indicate that the physical interaction between the two proteins is relevant to the control of lytic gene expression in dually infected cells.

The mechanism of the mutual inhibition is not completely clear yet. However because both K-RTA and EBV-Z require multimerization for their proper functions [75], [79], we suspect that the physical interactions between the two may affect their respective multimerization process and thus inhibit each others' function.

There is an interesting phenomenon referred as selective switch from latency to lytic replication in dually infected cells [12], [53]. The mechanisms behind the selective induction of lytic replication is unknown, but the phenomenon clearly suggests that viral factor(s) is involved in this selective induction [53]. Based on the facts that K-RTA and EBV-Z are the critical lytic replication initiators for KSHV and EBV latencies respectively, and our data that K-RTA and EBV-Z mutually inhibit each other's transactivations, we hypothesize that various chemical treatments and/or physiological stimuli may trigger differential expression of K-RTA or EBV-Z. The induced K-RTA and EBV-Z would neutralize each others' function through physical interactions. The predominantly-expressed gene product would block the function of another less-expressed one in dually infected cells. This process would result in only one critical lytic replication initiator remains functional in dually-infected PELs. The predominantly expressed molecule, either K-RTA or EBV-Z, would lead to the selective lytic replication of one virus in dually-infected PELs. The selective induction of lytic replication may facilitate the survival of the winning virus by maximally utilizing cellular resources.

However, KSHV is apparently has an advantage over EBV on the selective lytic replication processes. First, that the initiation of KSHV lytic gene expression correlated with the reduction of EBV lytic gene expression (Fig. 6) suggests that the presence of KSHV lytic genes is overriding the EBV lytic gene expressions at least in BC1 cells (Fig. 6A). Second, TPA has been shown to induce lytic replication of EBV in BC1 cells. We have found that TPA induces delayed expression of K-RTA, comparing to butyrate or TPA plus butyrate (Fig. 6B). The delayed induction of K-RTA may provide an opportunity for lytic replication of EBV in BC1 cells. In addition, the faster induction of K-RTA by TPA plus butyrate (Fig. 6B) provides an explanation that TPA and butyrate together induce KSHV, rather than EBV, lytic replication in BC1 cells. Third and finally, the induction of expression of the EBV-Z and E-RTA needs de novo protein synthesis while the induction of K-RTA of KSHV is not [80]. Therefore with all available data, KSHV is apparently having an advantage over EBV for the induction of lytic replication in dually infected PELs.

It is of note that the mutual inhibition of K-RTA and EBV-Z may be used by both viruses to maintain and/or establish dual latency in PELs. The dually infected cells are apparently more latent [53]. The mutual inhibition of the two lytic initiators may block some spontaneous lytic replications. In addition, the during primary infection processes to establish the dual latency, both KSHV and EBV lytic gene expressions are likely to be initiated [52]. The mutual inhibition of K-RTA and EBV-Z might play a role for the establishment of dual latency in the same cells in primary infection.

Our previous data suggest that EBV inhibits the lytic replication of both KSHV and EBV through another mechanism involved with EBV latent membrane protein 1 [52]. The general consequences of EBV-Z or LMP-1-mediated inhibition of KSHV lytic replication are different: LMP-1 inhibits both EBV and KSHV lytic replications, while EBV-Z only inhibits KSHV lytic replication, but initiates EBV lytic replication. The comparative studies of EBV-Z and LMP-1 for their inhibitory effects on KSHV lytic replication have not been carried out extensively. However, the new mechanism identified in this report may reinforce the point that two viruses would prefer to maintain respective latency in a dually infected cells. One explanation would be that the lytic replication of the viruses may lead to the eventual death of the host cells. Therefore, both viruses may use their interactions to block potential lytic replication inductions.

In summary, we have addressed how the two viruses interact with each other in dually infected PELs. Our data may provided a possible mechanism for maintaining viral latency and for selective lytic replication in dually infected PELs, *i.e*., through mutual inhibition of two critical lytic replication initiators. Of note, the majority of the current studies on lytic replications of KSHV and EBV are using KSHV or EBV single-infected cells as model systems. Therefore, there is a concern about the applicability of these studies in dually-infected PELs. Our data about putative interactions between EBV and KSHV would be applicable to the majority of AIDS-associated PELs and may be relevant to the pathogenesis of PELs.

## Materials and Methods

### Plasmids and antibodies

K-RTA, EBV-Z and E-RTA expression plasmids, K14A-luc and Pan-luc were described previously [18], [52]. Flag-EBV-Z expression plasmid was a gift from Dr. Paul Lieberman. The mutant plasmids were made with the proper primers and the use of Quick Change II Site-Directed Mutagenesis Kit (Stratagene). The oligonucleotides, 5′-CCACCGGCAAGGTCACTGGAAGCCAGTTTGTCATTAGCAAACCC-3′ and its complementary strand were used for deletion of the leucine heptapeptide repeat (LR) (a.a.# 246-292) in K-RTA (K-RTA-DLR). Primers, 5′-GCCGGGCCAAGTTTAAGCAACTGTGCCCAAGCCTGGATGTTGACTCC-3′ and its complement strand were used to delete the leucine zipper region (a.a.# 197-221) of EBV-Z (EBV-Z-DL). Primes, 5′-CAAATCATCTGAAAATGACAGGGATCGCCTCCTGTTGAAGCAGATG-3′ and its complement were used to change leucine at a.a. #214 of EBV-Z to aspartic acid (EBV-Z-L214D). CMV-β-galactosidase expression plasmid was from Clontech. Peptide antibody against K-RTA was described [52]. K8 antibody was from Dr. Jae Jung. EBV-Z monoclonal antibody (BZ1; sc-53904) and GAPDH (sc-47724) were purchased from Santa Cruz Biotechnology. Monoclonal EA-D (EBV-018-48180) was from Capricorn. E-RTA (11-008) antibody was from Argene. FLAG (F3165) and Tubulin (T6557) antibodies were purchased from Sigma. Cy-2-conjugated donkey anti-mouse IgG (715-225-150) and Cy5-conjugated donkey anti-rabbit IgG (711-175-152) antibodies were purchased from Jackson ImmunoResearch Laboratory.

### Cell Culture and Recombinant Adenovirus Infection

Akata (EBV+,KSHV−) is an BL line. BC1 (EBV+,KSHV+) and BC3 (EBV−, KSHV+) are PEL lines [81], [82]. These cells were maintained in RPMI1640 plus 10% FBS. 293T (EBV−,KSHV−) is human fibroblast line. 293EBV (EBV+, KSHV−) is a 293 fibroblast derived cell line with wild type EBV genome[38]. BRLF1KO and BZLF1KO were also 293 fibroblast derived cell lines with BRLF1 or BZLF1 deletion in their respective EBV genomes [38]. These three lines were maintained in DMEM plus 10% FBS plus 0.5 mg/ml hygromycin. AGS-BX11g is an epithelial cell line with EBV genome and was maintained in DMEM plus 10% FBS plus 0.5 mg/ml G418 [83]. The recombinant adenovirus for green fluorescence protein (GFP) (AdGFP) and K-RTA (AdRTA) were a gift from Dr. Byrd Quinlivan [84]. The recombinant adenoviruses were titered in 293 cells. AGS-BX11g cells were infected by recombinant viruses at a multiplicity of infection (MOI) of 10 (calculated from PFU). One day later, cells were then treated with TPA for induction of EBV lytic replication.

### Induction of lytic replication

12-*O*-tetradecanoylphorbol-13-acetate (TPA; from Sigma or Aldrich) was used to treat BC3 (5–10 ng/ml), BC1 (1–40 ng/ml), and AGS-BX11g (0.5–2 ng/ml) for induction of lytic replication. Sodium butyrate was also used for induction of lytic replications. Goat anti-human immunoglobulin G (IgG) (Sigma; Cat# I-9384) was used to activate EBV lytic replication in Akata cells. For immunostaining and co-immunoprecipitation experiments in Fig. 3, BC1 cells were treated with 10 ng/ml TPA overnight, and then treated with butyrate (0.5 mM). The cells were collected a day later for immunostaining and immunoprecipitation experiments.

### Transient Transfection, Isolation of Transfected cells, and Reporter Assays

Effectene (Qiagen) was used for the transfection of 293EBV, BRLF1-KO, BZLF1KO and 293T cells. Transfection of Akata cells were achieved by using Amaxa Nucleofector Device. Briefly, 5 µg of plasmids were transfected into 2×10^6^ cells in 100 µl solution V using program G016. Six hours later, the transfected cells were treated with anti-human-IgG. Transfection efficiency was about 70%. Electroporation (320V; 925 µF) was used for transfection of BC3 cells and the selection of transfected cells was essentially the same as described previously [85]–[87]. CD4 and other expression plasmids were transfected into BC3 cells. One day after the transfection, the cells were used for isolation of CD4-positive cells with the use of Dynabeads CD4 (Dynal Inc). The enriched cells were detached from the Dynabeads CD4 by incubation for 45–60 minutes at room temperature with 10 µl of DETACHaBEAD (Dynal). The detached beads were removed by using a magnet separation device. The released cells were washed 2–3 times with 500 µl RPMI 1640 plus 10% FBS, and resuspended in RPMI 1640 plus 10% FBS at 5×10^5^ cells/ml. Cells were split into two wells and recovered for 2–6 hours: TPA (5 ng/ml) were added into one well. The treated cells were collected one day later. The luciferase assays were performed using the assay kit from Promega according to manufacturer's recommendation.

### Western Blot Analysis, RNA extraction, and RNase Protection Assays (RPA)

Standard western blot analysis was performed as described [88]. Total RNA was isolated from cells using the RNeasy Total RNA Isolation Kit (Qiagen). RPA was performed with total RNA using the RNase Protection Assay Kit II (Ambion) as described [87]. The GAPDH probe was from US Biochemicals, Inc. The probe for K-RTA was made by PCR of the K-RTA region (BC1 coordinates: 72601-72900) followed by cloning into pcDNA3 vector.

### Co-immunoprecipitation (Co-IP)

BC-1 cells were treated with 10 ng TPA per ml for 24 h, and with 0.5 mM sodium butyrate for another 24 h. 293T cells grown in 10-cm plates were transfected with the designated plasmids, and cells were ready for experiments 24 hours later. These cells were washed with PBS and lysed at 4°C for 30 min in EBC buffer (50 mM Tris-HCl, pH7.5, 120 mM NaCl, 0.5% NP-40) supplemented with protease inhibitor cocktail tablet (Roche) with gentle rotation. The cell lysates were centrifuged at 16,100×g for 15 min, and the supernatants were recovered. For coimmunoprecipitation, lysates were pre-cleared with normal rabbit or mouse IgG with 20 ml Protein G Sepharose. Specific antibodies and Protein G Sepharose (GE Healthcare) at 4°C for 1hour or overnight. The beads were washed three times with EBC buffer, boiled in SDS loading buffer, and subsequent Western blot were essentially the same as described.

### Immunochemical analysis

The TPA and butyrate treated BC1 cells were centrifuged and washed with phosphate-buffered saline solution (PBS), and fixed with 4% paraformaldehyde for 15 minutes. The cells were permeabilized with 100% cold methanol for 5 minutes. After washing with PBS, the cells were blocked with PBST including 1% BSA for 30 minutes. The cells were incubated with antibodies against EBV-Z (1∶50 dilution) and K-RTA (1∶50 dilution) for one hour. Following three washing with PBST, the cells were incubated for one hour with Cy-2 conjugated secondary antibodies against mouse IgG (1∶60) and Cy5 -conjugated secondary antibodies against rabbit IgG (1∶60). Finally, DAPI was used for nuclei staining and the cells were mounted (Gel Mount Aqueous Mounting Medium, Sigma) in the poly-prep slides (Sigma) for analysis with confocal microscopy (Olympus FV500) in the Microscopy Core facility at the University of Nebraska-Lincoln.
